# Editorial: Beneficial Microbiota Interacting With the Plant Immune System

**DOI:** 10.3389/fpls.2021.698902

**Published:** 2021-06-22

**Authors:** Ioannis A. Stringlis, Paulo J. P. L. Teixeira, Roeland L. Berendsen, Corné M. J. Pieterse, Christos Zamioudis

**Affiliations:** ^1^Plant-Microbe Interactions, Department of Biology, Science for Life, Utrecht University, Utrecht, Netherlands; ^2^Department of Biology, 'Luiz de Queiroz' College of Agriculture (ESALQ), University of São Paulo (USP), Piracicaba, Brazil; ^3^Laboratory of Plant Pathology, Department of Agricultural Development, Democritus University of Thrace, Orestiada, Greece

**Keywords:** microbiome, beneficial microbes, crop protection, rhizosphere, phyllosphere, plant immune system, induced systemic resistance, plant pathogens

## Introduction

The Green Revolution during the 50s and 60s was a milestone in the history of mankind. Based on the principles “higher yields, more food, less poverty and hunger,” it radically transformed agriculture and dramatically increased global food production (Khush, 2001). Despite the success, intensive agricultural practices that include the exhaustive use of synthetic fertilizers and agrochemicals and the overexploitation of natural resources, eventually came with serious environmental costs (Tang et al., 2021). Today, more and more farmers around the world realize that the soils used to cultivate monocultures for many years are rapidly degrading (Banwart, 2011). In addition, the withdrawal of agrochemicals from the market that are effective but unsafe for the environment and the consumer health created additional difficulties in the control of devastating pathogens and pests. With the advent of a rapidly growing human population, anticipated to reach about 10 billion people by the year 2050 (FAO, 2009), a new revolution in agriculture seems to be more timely than ever in order to sustain and further increase food production (Evans and Lawson, 2020).

Plants are massively colonized by communities of microbes that are referred to as the plant microbiota. Plant-associated beneficial microbes have long been known to provide important ecosystem services and promote plant health by enhancing growth, suppressing pathogens and training plant immunity (Berendsen et al., 2012; Trivedi et al., 2020). Lorenz Hiltner, a pioneer of microbial ecology, was the first to recognize the important role of beneficial bacteria that colonize the rhizosphere (Hartmann et al., 2008). Since then, our understanding regarding the structure and the function of the plant microbiomes has been greatly improved (Tian et al., 2020). Towards a new Green Revolution that is protective to the environment and safe to humans, the enhanced interest in the plant microbiome clearly stems from its strong potential to provide eco-friendly solutions in plant disease protection and novel tools to promote sustainability in agroecosystems (Qiu et al., 2019).

Understanding the complexity of plant-microbiome interactions is essential to transform fundamental knowledge to microbiome-informed innovations in modern agriculture. We host here in this Research Topic, “Beneficial Microbiota Interacting with the Plant Immune System,” 16 articles that enhance our knowledge on the supportive functions of beneficial microbes in plant disease resistance. In particular, the Topic contains research articles focusing on the protective functions of individual biological control agents (BCAs) against important diseases in agricultural and forest ecosystems, but also metagenomic studies that provide a more holistic view on the way microbiota interact with plant immunity. In addition, one method paper presents a pipeline to dissect selected plant responses to bacteria with different lifestyles and 5 review articles summarize our current knowledge on the mechanisms by which beneficial bacteria and fungi promote host defenses and plant health in below- and aboveground plant tissues. New experimental platforms and integrated approaches that combine (meta)-omics with functional analyses are needed in future research in order to obtain a comprehensive understanding of the mechanisms by which beneficial microbes interact with phytopathogens and plant immunity.

## Content Collection

### Original Research Articles

By testing more than 500 bacterial isolates, Park et al. identified *Bacillus thuringiensis* strain JCK-1233 and a specific diketopiperazine produced by JCK-1233 to induce resistance in pine trees thereby suppressing the wilt disease caused by the nematode *Bursaphelenchus xylophilus*, one of the most important pests affecting pine forests worldwide. Thus, also in forest ecosystems where the application of synthetic pesticides against devastating pests is costly and often complicated by diverse ecological risks, BCAs could be implemented as eco-friendly and cost-effective alternatives.

Chen et al. studied the mechanisms by which another Bacillus strain, *Bacillus velezensis* CLA178, suppresses the tumors caused by Agrobacterium infection in the ornamental plant *Rosa multiflora*. Combined with work in Arabidopsis, the authors found that, when applied to the roots, CLA178 promoted plant growth and further primed the expression of defense genes regulated by the salicylic acid and ethylene signaling pathways. The sequenced genome of CLA178 included in the study pave the way for genome-centered future analyses in order to reveal the bacterial genetics involved in both phenomena.

Wu et al. utilized pot experiments to demonstrate that the antagonism between the biocontrol strain *Bacillus velezensis* HN03 and the wilt pathogen of banana *Fusarium oxysporum* f. sp. *cubense*, depends on the nutritional content of the soil. In particular, the authors found that synchronous application of HN03 and compost potentiated the biocontrol outcome of individual treatments through reciprocal interactions, which the authors summarize in a conceptual model. Considering that the application of BCAs in the field often fails to deliver the anticipated outcomes, this study provides means to enhance the biocontrol activity of selected BCAs under agricultural settings.

In their genomics study, Samaras et al. provide insights into the mechanism underpinning the protective functions of *Bacillus subtilis* MBI 600, one of the many commercialized *Bacillus* strains. The authors sequenced the MBI 600 genome and through comparative genomics identified common genes to other Bacilli, but also unique genomic features related to root colonization, plant growth promotion and biocontrol activity. Interestingly, by generating an *yfp*-tagged strain, the authors were able to study the colonization potential of MBI 600 in the roots of cucumber in different growth substrates. Overall, this interesting study demonstrates the power of microbial genetics in dissecting the mechanisms by which beneficial microbes train immunity and improve host health.

Sacristán-Pérez-Minayo et al. characterized the effects of two *Pseudomonas* strains on sugar beet productivity. When used as soil inoculants in the field, both beneficials improved the yield and the quality of the tubers, however, they failed to provide protection against two important sugar beet pathogens. The study clearly suggests that delivering multiple traits in the field could be a difficult task; rather than the application of single microbes, the application of microbials at the community level may be a more reasonable approach.

In their study, Chen et al. demonstrated that prolonged monoculture with the medicinal herb *Radix pseudostellariae* reduced the diversity of antagonistic *Trichoderma* communities in the rhizosphere, consequently increasing the abundance of pathogenic *Fusarium oxysporum*. Interestingly, the authors found that the application of the *Trichoderma* strain *T. harzianum* ZC51 could improve plant resistance and reduce the growth inhibitory effect stemming from the consecutive monoculture. This study clearly shows the impact of monoculture on the rhizosphere microbiome and further provides means to improve soil health through the application of soil beneficials.

The interesting work of Anguita-Maeso et al. reveals that a wild olive variety that is otherwise resistant to the wilt fungus *Verticillium dahliae* becomes susceptible to the pathogen when propagated *in vitro*. The authors provide evidence that *in vitro* micropropagation of this particular olive accession alters community structure resulting in the breakdown of resistance to *Verticillium*. Thus, the xylem microbiomes could be exploited as a reliable resistance source to devastating root-infecting pathogens.

The seedling stage is the most vulnerable time in the life cycle of a plant, and the role of seed-derived microbiota in promoting seedling health is well-established. Focusing on rice, Wang et al. utilized an axenic growth system and carried out metagenomic analyses to demonstrate that during seed germination, the pool of microbes that colonize the seeds are separated to distinct assemblages in the different plant tissues. Interestingly, the authors found that functions related to plant growth and pathogen suppression are enriched in the core microbiomes transferred from the seed to the newly established plants.

Invasive alien plant species (IAPS) may cause severe damage to natural ecosystems by reducing the richness and abundance of native plant species. *Mikania micrantha*, a fast-growing vine, is ranked amongst the top 100 worst IAPS in the world. Having the authors previously published the *M*. *micrantha* genome, the exciting metagenomics study of Yin et al. in this collection indicates that the roots of *M*. *micrantha* host more phosphate-solubilizing and pathogen-suppressive rhizobacteria than the roots of two coexisting native plant species. Thus, rhizosphere microbes seem to play important roles in the establishment of invasive plants and may even act as drivers of plant invasion.

The leaves of the perennial herb *Tricyrtis macropoda* have an unusual phenotype with spots covering the leaf surface. Wang et al. found that the composition of the fungal microbiome in the spots differs from the fungal communities in the green parts. By analyzing the metabolome of spotted and non-spotted leaf parts, a significant correlation between the endophytic fungal communities and the production of metabolites has been established. Overall, this study provides new insights into the relationship between microbes and plant phenotypes and further demonstrates the value of -omics toward understanding the molecular cues driving microbiome assembly in the host.

### Methods Articles

Hydrogen peroxide (H_2_O_2_) functions as an important signaling molecule in plants during biotic interactions. Carril et al. developed a protocol to visualize and quantify H_2_O_2_ production in wheat leaves after infection with a pathogenic bacterium or after co-inoculation of the pathogen with a beneficial bacterium. DAB staining combined with an imaging analysis pipeline revealed that co-inoculation yielded less H_2_O_2_ accumulation and less visible disease symptoms compared to the pathogen infection alone. Therefore, this protocol can successfully determine the H_2_O_2_ levels accumulating in response to bacteria with different lifestyles.

### Review Articles

A fast-growing field of research focuses on microbial biocontrol in the phyllosphere. Legein et al. review the different factors influencing microbial adaptation in the phyllosphere. These factors range from environmental stresses to microbe-microbe and plant-microbe interactions. The authors present the current knowledge on the interplay between these factors and dissect the mechanisms involved in the biocontrol activity of microbial inoculants in the phyllosphere. Demonstrating examples from *in vitro* and field experiments, the authors suggest the integration of experimental data coming from both sources to design successful and sustainable biocontrol strategies against leaf pathogens.

Our knowledge on the plant microbiome and the diverse ways it affects plant fitness is expanding continuously. This is also demonstrated in the review by Lee and Ryu, where algae are presented as new members of the beneficial plant microbiome. The authors discuss their presence on plant tissues and in the soil and their occurrence in plant microbiome datasets. They further provide examples highlighting the beneficial effects of algae on plant growth and disease suppression as well as the mechanisms involved. Application of algae as biofertilizer to modulate microbiome activity and improve crop yields is suggested as an extra alternative to conventional agricultural practices.

Focusing on the three major classes of soil-borne beneficials, plant growth promoting rhizobacteria, biological control agents and root nodulating rhizobia, Lucke et al. provide an excellent overview of the mechanisms by which these classes of microbes deliver plant-growth and disease-suppressive compounds. Toward identifying potent biologicals, the authors point to the need for genome-centered studies in order to identify the microbial genes responsible for the beneficial functions.

Ectomycorrhizal fungi (EMF) are soil-borne microbes that form mutualistic associations with forest trees. The review of Dreischhoff et al. brings into the light a thus-far unexplored function of the EMF related to plant immunity. The authors review the evidence supporting that EMF activate local and systemic immune responses with the latter sharing characteristics of both induced and systemic acquired resistance. Toward enhancing our understanding of the mechanisms by which EMF interact with the immune system of host trees, the authors provide a guide to future research that will help to reveal aspects related to EMF-induced resistance.

The ability of beneficial microbes to modulate plant immunity largely relies on the secretion of a diverse array of low-molecular weight metabolites. The molecular determinants specifically involved in the phenomenon of rhizobacteria-mediated induced systemic resistance is reviewed in this article collection by Pršić and Ongena. The authors provide an updated overview of ISR elicitors originating from diverse rhizosphere bacterial species, such as acyl-homoserine lactones, cyclic lipopeptides, rhamnolipids, N-alkylated benzylamine derivatives, siderophores, antibiotics, and volatile organic compounds. They further emphasize on the necessity to reveal in future studies how these molecules are sensed by the host and what type of defense responses are manifested upon elicitor perception.

## Author Contributions

The authors jointly defined the content of this Research Topic and participated in the editing process. All authors made substantial, direct and intellectual contribution to the composing of this editorial, and approved it for publication.

## Conflict of Interest

The authors declare that the research was conducted in the absence of any commercial or financial relationships that could be construed as a potential conflict of interest.

## References

[B1] Banwart S. (2011). Save our soils. Nature 474, 151–152. 10.1038/474151a 21654781

[B2] Berendsen R. L. Pieterse C. M. J. Bakker P. A. H. M. (2012). The rhizosphere microbiome and plant health. Trends Plant Sci. 17, 478–486. 10.1016/j.tplants.2012.04.001 22564542

[B3] Evans J. R. Lawson T. (2020). From green to gold: agricultural revolution for food security. J. Exp. Bot. 71, 2211–2215. 10.1093/jxb/eraa110 32251509

[B4] FAO (2009). How to Feed the World in 2050. High Level Expert Forum. FAO, Rome.

[B5] Hartmann A. Rothballer M. Schmid M. (2008). Lorenz Hiltner, a pioneer in rhizosphere microbial ecology and soil bacteriology research. Plant Soil 312, 7–14. 10.1007/s11104-007-9514-z

[B6] Khush G. S. (2001). Green revolution: the way forward. Nat. Rev. Genet. 2, 815–822. 10.1038/35093585 11584298

[B7] Qiu Z. Egidi E. Liu H. Kaur S. Singh B. K. (2019). New frontiers in agriculture productivity: optimised microbial inoculants and *in situ* microbiome engineering. Biotechnol. Adv. 37:107371. 10.1016/j.biotechadv.2019.03.010 30890361

[B8] Tang F. H. M. Lenzen M. McBratney A. Maggi F. (2021). Risk of pesticide pollution at the global scale. Nat. Geosci. 14, 206–210. 10.1038/s41561-021-00712-5

[B9] Tian L. Lin X. Tian J. Ji L. Chen Y. Tran L.-S. P.. (2020). Research advances of beneficial microbiota associated with crop plants. Int. J. Mol. Sci. 21:1792. 10.3390/ijms21051792 32150945 PMC7084388

[B10] Trivedi P. Leach J. E. Tringe S. G. Sa T. Singh B. K. (2020). Plant–microbiome interactions: from community assembly to plant health. Nat. Rev. Microbiol. 18, 607–621. 10.1038/s41579-020-0412-1 32788714

